# The Difficulties and Needs of Organ Transplant Recipients during Postoperative Care at Home: A Systematic Review

**DOI:** 10.3390/ijerph17165798

**Published:** 2020-08-11

**Authors:** Fu-Chi Yang, Hsiao-Mei Chen, Chiu-Mieh Huang, Pei-Lun Hsieh, Shoei-Shen Wang, Ching-Min Chen

**Affiliations:** 1 College of General Education, National Chin-Yi University of Technology, Taichung 41170, Taiwan; caring686868@gmail.com; 2 Institute of Allied Health Sciences, College of Medicine, National Cheng Kung University, Tainan 701401, Taiwan; 3 Department of Nursing, Chung Shan Medical University, Taichung 40201, Taiwan; fionajunlom@gmail.com; 4 Institute of Clinical Nursing, School of Nursing, National Yang-Ming University, Taipei 11221, Taiwan; cmhuang@ym.edu.tw; 5 Department of Nursing, College of Health, National Taichung University of Science and Technology, Taichung 40343, Taiwan; peilun@nutc.edu.tw; 6 Department of Surgery, National Taiwan University Hospital, Taipei 100225, Taiwan; wangp@ntu.edu.tw; 7 College of Medicine, National Taiwan University, Taipei 100233, Taiwan; 8 Department of Surgery, Fu Jen Catholic University Hospital, New Taipei City 24352, Taiwan; 9 School of Medicine, Fu Jen Catholic University College of Medicine, New Taipei City 242062, Taiwan; 10 Department of Nursing, Institute of Allied Health Sciences, College of Medicine, National Cheng Kung University, Tainan 701401, Taiwan

**Keywords:** transplantation, postoperative care, difficulties, needs, nursing, systematic review

## Abstract

With recent advances in surgery and immunosuppressive drugs, organ transplantation has become a major treatment for irreversible organ failure. However, organ transplant recipients returning home after operation may face ongoing physiological, psychological, and social difficulties. To increase recipients’ quality of life, postoperative care at home is critical. Thus, the aim of this systematic literature review was to explore recipients’ difficulties and needs during postoperative care at home. Our search conformed to Preferred Reporting Items for Systematic Reviews and Meta-Analyses (PRISMA) guidelines and returned 23 relevant articles published from 1997–2020 in PubMed, MEDLINE, EBSCO, Cochrane, ProQuest, and CEPS, which were assessed using the Modified Jadad Scale or the 32 Consolidated Criteria for Reporting Qualitative Research (COREQ) appraisal indices and then synthesized through narration. The most common difficulties faced were psychological difficulties, followed by physiological, social, and other difficulties; the most common needs were psychological needs, followed by education and information training, social, and other needs. These results demonstrated that healthcare professionals can do more to provide patients with comprehensive care and promote successful self-management and quality of life at home. They also confirmed that collaboration between transplant teams, caregivers, and patients is necessary to optimize postoperative outcomes. We suggest that customized care may promote postoperative patients’ self-management and quality of life at home.

## 1. Introduction

With the advances in surgery and immunosuppressive drugs, organ transplantation—the epoch-making breakthrough in modern surgical science—has become one of the major treatments for irreversible organ failure, as it can prolong patients’ lives and improve their quality of life [1,2]. Organ transplantation is currently the most effective method for treating end-stage organ failure, enabling patients on the verge of death to continue living [3]. Organ transplantation refers to the removal of all or part of a body organ or tissues and the provision of these to suitable patients experiencing organ failure. It is performed to treat diseases and save lives, thereby improving patients’ quality of life and prolonging their life expectancy [4].

Every transplant organ must go through a series of evaluation, matching, screening, and waiting procedures before it can be used in the transplant surgery; as such, it is often difficult for patients to secure organs and transplant surgeries. During the organ transplantation process, patients often undergo complicated physiological, psychological, social, and spiritual dark-recovery experiences, requiring close assistance from their healthcare team [5]. Healthcare teams should pay attention not only to patients’ physical conditions but, also, to their mental states in order to improve their overall prognosis.

Although organ transplantation enables patients to live longer, they may still face the postoperative difficulties of infections, organ rejections, and even death. The previous literature has noted that, owing to the need for the long-term use of immunosuppressive drugs after surgery, transplant recipients face many physical, mental, and social difficulties caused by side effects after surgery, and patients may encounter many comorbidities during postoperative long-term care [6]. However, the needs of transplant recipients and the difficulties they face when they return home are often ignored by healthcare professionals [7]. Difficulties and needs can present barriers to successful treatment; as such, a deeper understanding of patients’ perceived barriers is needed to develop better interventions.

Although transplantation gives patients a new chance to live, the process entails an emotional roller coaster. Even after the psychological torture of the waiting period and the joy following a successful transplantation, various postoperative problems can cause physical and mental difficulties for patients, such as taking on the risks of transplant surgery, acute and chronic rejection of organs, adherence to medication regimens that must be strictly followed throughout life, lingering side effects of immunosuppressive agents, adaptation and maintenance of new organs, uncertainty about the future, and renewal of new organs [8].

Needs arise as the result of judgment from personal, subjective cognition. The perception of a lack or insufficiency impels an individual to act in order to meet these needs. Meeting needs can relieve or reduce an individual’s level of anxiety or annoyance, improve their feelings about their current situation, and increase comfort and well-being [9]. If difficulties are the problems that patients face after discharge, then needs allude to the solutions that could potentially solve these problems. Therefore, healthcare professionals should care for the difficulties and needs of postoperative transplant recipients at home.

Organ transplantation is a relatively new breakthrough in the rapid advancement of modern surgical medicine, and it not only prolongs the lives of patients but, also, enables them to regain their health and happiness. Nonetheless, complex physiological, psychological, social, and spiritual issues arise for patients in the process of organ transplantation [10]. Transplant recipients and their families require close assistance from medical teams. Therefore, the aim of this systematic review is to empower medical teams to provide specific assistance sooner by locating literature related to the difficulties and needs faced by transplant recipients who are receiving postoperative care at home.

## 2. Materials and Methods

This study involved a systematic review that did not use a review protocol. The databases of PubMed, MEDLINE, EBSCO, Cochrane, ProQuest, and CEPS were used, with the following inclusion criteria for studies: (1) participants were organ transplant recipients, and (2) the study results revealed the difficulties and/or needs of organ recipients during the process of postoperative care at home. The exclusion criteria were studies examining participants that were (1) organ donors, (2) the primary caregivers of organ transplant recipients, and (3) medical staff involved in organ transplantation. The search terms were “transplant recipients” AND “difficulties” OR “difficulty”, “adjustment” OR “Quality of Life” OR “needs” AND “postoperative home care”, and “recipients” AND “difficulties” OR “difficulty” OR “adaptation” OR “quality of life” OR “needs” AND “home care process.”

**Figure 1:**
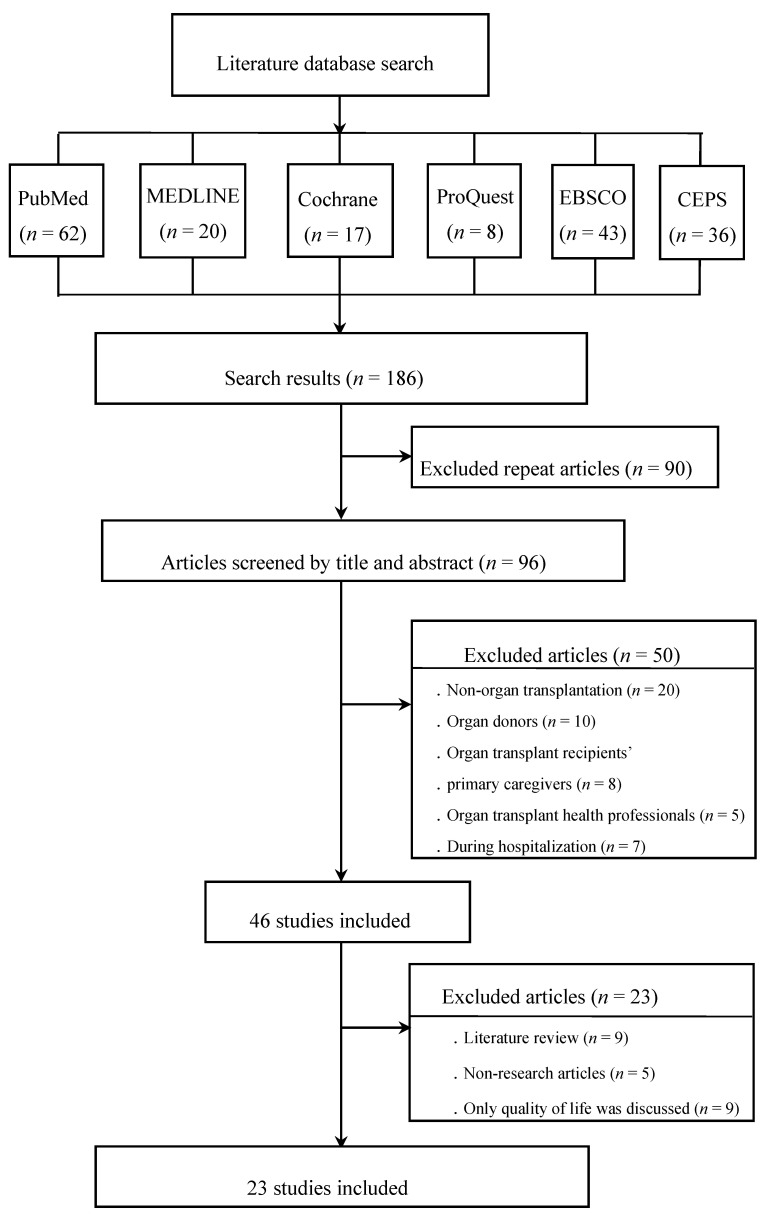
Preferred Reporting Items for Systematic Reviews and Meta-Analyses (PRISMA) flow diagram: results of the literature search and selection.

Only published articles written in English or Chinese that discussed research involving humans were included. Since there were insufficient articles available on this topic before 1997, the date range was extended from January 1997 to January 2020. This review conforms to the Preferred Reporting Items for Systematic Reviews and Meta-Analyses (PRISMA) guidelines [11]; see Table S3. The PRISMA flow diagram is shown in Figure 1.

### 2.1. Search Strategy and Outcomes

Relevant articles were gradually retrieved based on the database characteristics and the complete search strategy—which involved examining the research aim, methods, research design, level of evidence, quality of research, and results for each article—to ensure the integrity of the literature retrieval. A total of 186 articles were retrieved—at which point, we read each article manually and deleted duplicate and unqualified articles. In the end, 23 articles were selected for inclusion.

### 2.2. Quality Appraisal

We reviewed 16 quantitative and 7 qualitative studies at the outcome level. We used the Modified Jadad Scale [12] to assess the quality of the quantitative studies and the Consolidated Criteria for Reporting Qualitative Research (COREQ) [13] to assess the quality of the qualitative studies. Three researchers independently assessed the quality of the studies.

The Modified Jadad Scale includes eight questions (total scores range from −2–8 points): (1) whether there is a description of randomization, (2) whether the randomization is appropriate, (3) whether there is a description of blinding, (4) whether the blinding is appropriate, (5) whether there is a description of withdrawals and dropouts, (6) whether there is a description of inclusion and exclusion criteria, (7) whether there is a description of the evaluation process for adverse reactions, and (8) whether there is a description of the statistical analysis methods. Higher scores indicate better research quality [14]. Among these 16 quantitative studies, one study (6.3%) scored 1, four studies (25%) scored 2, eight studies (50%) scored 3, two studies (12.5%) scored 4, and one study (6.3%) scored 6. Most studies used purposive sampling instead of random assignment, and no double-blind trials were performed, showing that the quality of the research designs could be strengthened and that there might be selection bias. The quality scores of each study are shown in Table S1.

COREQ, developed by Tong, Sainsbury, and Craig [13], includes 32 appraisal indices (Table S2). Using the indices most relevant to the contents of each study, we found that (1) the researchers of the 7 qualitative studies all had a nursing background, (2) the participants were all transplant recipients, (3) purposive sampling was used in all studies, (4) the number of participants ranged from 8 to 41, and (5) most data were collected through interviews. One of the studies collected retrospective data (within a limited time frame) in the form of posts from two discussion threads on the International Transplant Community website.

### 2.3. Data Extraction and Synthesis

In addition to the information we extracted to perform the quality assessments (regarding methods, interviewer information, samples, and so on—see Tables S1 and S2), we extracted information relating to transplant patients’ difficulties and needs during postoperative care at home. This included details about patients’ symptoms, emotions, fears, relationships, strategies, and resources. For qualitative studies, participants’ care needs and difficulties were identified through the themes deduced from a content analysis. Finally, we employed a systematic review approach, because the included studies had varied results; thus, the findings are presented through narration.

## 3. Results

Twenty-three research articles were obtained through our complete systematic review strategy (Figure 1 and Table A1). The 23 articles were from 15 countries, and they were published between 1997 and 2020. The countries included Germany (*n* = 1), the Netherlands (*n* = 1), Sweden (*n* = 2), Switzerland (*n* = 2), Norway (*n* = 1), Spain (*n* = 1), the United Kingdom (*n* = 1), Turkey (*n* = 1), the United States (*n* = 5), Canada (*n* = 1), Brazil (*n* = 1), Australia (*n* = 1), Japan (*n* = 1), China (*n* = 2), and Taiwan (*n* = 2). All included articles were summarized to present a complete understanding of the transplant recipients’ postoperative care at home (Table A1).

### 3.1. Research Aim and Participants

Most participants were kidney transplant recipients [7,15,16,17,18,19,20,21,22,23,24,25,26], followed by heart transplant recipients [5,27,28,29], liver transplant recipients [30,31], and lung transplant recipients [32]. Three studies were not limited to specific organ transplant recipients [33,34,35]. One study focused on adolescents and pediatric recipients [18]. Eight studies assessed the psychological adjustment of organ transplant recipients [7,16,18,20,21,30,31,34], eight studies assessed both the quality of life and other aspects of transplant recipients’ experiences [22,23,24,26,28,29,33,35], two studies assessed the sleep quality of transplant recipients [15,25], and six studies assessed patients’ experiences and coping strategies, alongside the assistance they needed during the dark-recovery stage [5,17,19,27,28,32].

### 3.2. Difficulties Faced by Recipients

With the advances in and prevalence of organ transplantation, the importance of postoperative care and the maintenance of patients’ quality of life cannot be ignored. However, the fact that organ transplant recipients must return home for self-care after surgery makes intervention during this period difficult. The physical, mental, and social barriers that recipients encounter during the post-operation transition are key issues that influence patients’ quality of life, potentially slowing or preventing their total recovery.

Based on our analysis of the 23 selected studies, the most common difficulties faced by transplant recipients in the process of postoperative care at home included (1) psychological difficulties, including anxiety or depression in response to uncertainty about the future, (2) physiological difficulties, such as physical discomfort after the transplantation, (3) social difficulties, including economic challenges, and (4) other difficulties, such as medical difficulties and difficulties faced by adult transplant recipients of different genders. These are summarized below.

#### 3.2.1. Psychological Difficulties

Recipients often faced various difficulties simultaneously. Among them, psychological difficulties were the most common. Eleven studies [5,7,16,19,21,25,26,28,31,32,33] reported the psychological difficulties that patients experienced, such as emotional and psychological problems owing to the fear of organ rejection, infection, and graft dysfunction. Nine studies [5,19,21,25,26,28,31,32,33] discovered that, after transplantation, patients experienced depression, distress, and anxiety, which influenced various aspects of their daily life and lowered their medical adherence. Causes of psychological difficulties included the stress of maintaining medical regimens, worries about the future, and fears of graft loss, as well as discomfort concerning medication side effects. Eight studies [5,7,16,19,21,26,28,31] found that, post-transplantation, patients often experienced mood swings based on their physical conditions, coped with emotional ups and downs, and experienced anxiety or depression concerning the uncertainty of the future and the threat of graft loss. Four studies [5,19,21,32] showed that patients experienced psychological recovery and emotional transition with the aid of various support sources.

#### 3.2.2. Physiological Difficulties

Nine studies [5,15,18,21,24,25,26,32,33] identified the physiological difficulties that patients experienced, including physical discomfort, pain, infection, fatigue, sleep disorders, various complications, and side effects from the long-term use of antirejection and immunosuppressive drugs. Multiple symptom distresses caused inconveniences, including rehospitalization and restrained daily activities, leading to a reduced quality of life. Patients reported challenges in symptom and pain management, which, in some cases, induced serious psychological difficulties.

#### 3.2.3. Social Difficulties

Six studies [7,16,18,21,31,32] explored the social difficulties experienced by transplant recipients. These studies noted that recipients may feel socially isolated because of their chronic physical condition, which may affect their self-image and relationships with others. After transplantation, patients need to adapt to a new lifestyle and social role. For example, to avoid infection, patients may need to limit their social contact with others. Four studies [24,25,31,33] noted that, owing to the considerable costs of the medical and surgical procedures, recipients faced economic difficulties that became a family burden post-transplantation.

#### 3.2.4. Other Difficulties

Other difficulties mentioned were medical difficulties [7,19,21], such as challenges managing one’s medical regimen. Four studies compared the distinct difficulties faced by men and women [16,24,28,30]. One study indicated that men felt more stressed about “interactions” and “uncertainty” than did women [16]. However, another study discovered that women had more difficulties with psychosocial adjustments than men did [30]. Wang et al. [24] specifically noted that male patients experienced impotence, which may, in some way, cast a shadow on their sense of masculinity. The difficulties faced by patients of different genders were notably distinct, which requires further investigation.

### 3.3. Needs Faced by Recipients

Beyond attending to the difficulties encountered by transplant patients at home, studies have found that healthcare professionals could improve patients’ quality of life by paying attention to their needs. Maslow, the father of humanistic psychology, believed that maintaining optimal health for humans involves meeting humans’ basic needs. His well-known theory, the Hierarchy of Needs, progresses from lower-level to higher-level needs: physiological (such as air, food, place to live, sleep, etc.); safety (such as sense of security, freedom from pain, and protection); love and belongingness (such as intimacy and friendship); esteem; and self-actualization [36]. Maslow asserted that higher-level needs could only arise when lower-level needs were met. Maslow’s theory helps clinical practitioners to assist patients with meeting their basic needs, such as by relieving their pain or treating their sleeping disorders.

Based on review of the 23 studies, the common needs faced by transplant recipients in the process of postoperative care at home included (1) psychological needs, (2) education and information training needs, (3) social needs, and (4) other needs.

#### 3.3.1. Psychological Needs

The most common needs noted by patients were psychological needs, as revealed in eight studies [5,7,20,22,27,29,30,34]. Following operation, emotional and spiritual support needs are particularly crucial for patients’ mental well-being. In addition to the physical pain after transplantation, recipients often experience negative emotions, which lead to further psychological needs. Psychological needs are common among patients, because patients rely on the help and care of others, a dependence that becomes pronounced when they are left alone.

#### 3.3.2. Education and Information Training Needs

Many patients believed that they did not obtain clear and necessary information before discharge. Six studies [7,17,18,23,31,35] discussed the need for education and information, which can help patients address various challenges after transplantation. Education and information are necessary to improve patients’ self-management.

#### 3.3.3. Social Needs

Five studies addressed patients’ social needs [7,19,22,27,28]. These found that patients sought social support from family, friends, and other patients post-operation. It is necessary to support patients in their interactions with friends and family to promote patients’ quality of life. One study [28] specified the need for tangible and financial support to address economic needs.

#### 3.3.4. Other Needs

Three studies [19,21,32] explored patients’ medical needs—for example, healthcare support to limit the impact of symptom distress. Two studies discussed different needs by gender [20,28]. One study [18] discovered that the needs of adolescents included information, coping strategies, and social support, which helped adolescent patients in their transition to adult healthcare. Future research should examine the needs of transplant recipients in different age groups.

## 4. Discussion

This systematic literature review of 23 studies explored the difficulties and needs of organ transplant recipients during postoperative care at home. Eleven studies showed that most transplant recipients experienced psychological difficulties [5,7,16,19,21,25,26,28,31,32,33], and five studies showed that recipients were susceptible to anxiety and depression [5,16,19,21,31]. One study also indicated that depressive symptoms could influence patients’ therapy adherence after kidney transplantation [37].

Other factors were also associated with postoperative care at home, such as gender-related factors—namely, the different difficulties faced by men and women [16,24,28,30]. In addition to gender differences, age differences should be considered when implementing effective postoperative care at home. One study discussed the difficulties faced by adolescent transplant recipients, which differed from those faced by adults [18]. Organ transplant recipients of different ages may thus have distinct difficulties that are worthy of attention.

The physiological difficulties encountered by patients post-operation, including impaired sleep quality, affect patients’ overall quality of life. This result echoes another study, which found that sleep quality is associated with key psychological indicators, such as depressive symptoms and psychological well-being [38]. Medical teams should therefore consider how to help organ transplant recipients overcome the multiple dark-recovery experiences they may face.

This systematic review discovered that patient needs for education, training, and information have not been sufficiently met [7,17,18,21,22,23,29,31,35]. Past studies have shown that supporting transplant recipients by increasing their disease-specific knowledge can produce medical and psychological benefits [39]. As such, healthcare professionals should arrange appropriate postoperative educational activities to help patients undergo the adjustment process of returning home.

This review found that psychological adjustment was patients’ most prevalent need [5,7,18,19,21,22,27,28,29,30,32,34], echoing the findings of previous research [40,41,42]. Furthermore, we found that the care needs of transplant recipients—including psychological needs, medical system and information needs, physiological needs, and so on—were not met through postoperative care at home. Many studies have shown that social needs are commonly reported by transplant recipients [7,18,19,21,22,27,28,29,32,34]. Therefore, it is vital to empower patients to obtain social support from family, friends, and medical team members in order to bolster their quality of life. In addition, healthcare providers should offer organ transplant recipients targeted counseling concerning postoperative care at home that considers patients’ psychological, spiritual, and social difficulties based on their age and gender.

Healthcare professionals should pay more attention to patients’ psychological difficulties post-transplantation to improve their quality of care. Healthcare professionals—as part of their role and as a way of completing their job—should involve themselves in this postoperative phase of the transplantation process to improve patients’ overall prognoses. Medical teams particularly need to equip transplant recipients to self-regulate and manage their self-care after they return home. Case-by-case counseling assistance should also be provided to assist patients, to improve the quality of professional care, and to serve as a reference for postoperative home care and future research.

### Limitations

At the study level, the conclusions reported in most of the quantitative studies included in our analysis may have reflected selection bias, as these studies used purposive sampling rather than random assignment and because none involved double-blind trials. At the review level, this systematic review was limited to literature from 1997 to 2020. The articles were only written in English, and only published articles were included. Therefore, this review may not fully or accurately represent the overall needs and difficulties of all organ transplant recipients.

## 5. Conclusions

Organ transplantation plays a vital role in patients’ survival, but the difficulties and needs experienced by transplant recipients during postoperative care at home also affects their recovery situation. This systematic literature review revealed the varied difficulties and needs of recipients post-transplantation, including how these difficulties generate further needs. Although this issue is critical for improving the treatment quality, it has rarely been discussed. Focusing on organ transplant recipients’ difficulties, needs, and the continuity of care in the postoperative period is beneficial for enhancing the quality of care that transplant recipients receive at home. Future research should expand on this study to isolate additional means of enhancing the quality of professional care.

We suggest that close cooperation between transplant teams, significant caregivers, and patients be established to meet patients’ needs post-operation. In addition, patients and families may benefit from post-transplantation psychological interventions to help them confront difficulties after returning home. Healthcare professionals should develop strategies based on patient needs, establish an Interprofessional Collaborative Practice care model, and provide tailored follow-up care to promote patients’ self-management and increase their quality of life at home.

## Supplementary Materials

The following are available online at https://www.mdpi.com/1660-4601/17/16/5798/s1: Table S1: Quality appraisal of included quantitative studies. Table S2: Quality appraisal of included qualitative studies. Table S3: PRISMA Checklist.

## Appendix A

**Table A1:** Descriptions of the included studies.

Author	Aim	Sample and Country	Study Design	Level	Quality	Main Results
1. Wang et al. [24]	To explore the symptoms and experiences of kidney transplant recipients and their relationship with antecedents and QoL	*n* = 294, aged 20–73 years. China.	A cross-sectional design.	II	3	Hair loss, concentration or memory problems, joint pain, difficulty seeing, and diarrhea were the top five severe symptoms. Males’ most troubling symptoms were impotence, cognitive decline, swollen glands, shortness of breath, and a changed sense of taste.
2. Klewitz et al. [17]	To explore satisfaction with information received about ISM among patients after kidney transplantation.	*n* = 440, aged > 16 years. Germany.	This cohort study had a cross-sectional design with an explorative approach.	II	2	Dissatisfaction was most notable for information concerning ISM side effects like drowsiness (57.1%) and depleted sex drive (56.3%). Older age, better adherence, greater perceived social support, and lower anxiety levels were correlated with higher satisfaction with ISM information.
3. Been-Dahmen et al. [7]	To understand kidney transplant recipients’ experienced self-management issues and necessary support.	*n* = 41, aged 31–69 years. The Netherlands.	A cross-sectional qualitative study.	III	N/A	(1) Recipients experienced postoperative difficulties in medical, emotional, and social tasks, such as adjusting to a new lifestyle and medical regimen, establishing relationships with nurses, dealing with social interactions and emotions, and improving their self-image. (2) Participants wanted to receive disease-specific knowledge, share personal experiences, consult their medical team, and feel encouraged.
4. Tamura et al. [22]	To assess recipients’ QoL after kidney transplantation and identify the relevant physical, mental, and socioeconomic factors.	*n* = 68, mean age = 51.7 ±13.5 years. Japan.	A multiple regression analysis.	III	3	Mood status—and especially confusion and fatigue—was a key factor in improving recipients’ QoL after transplantation. Continuous patient education, dissemination of stress-coping skills, and consistent psychological symptom screening can improve kidney recipients’ QoL.
5. Xie et al. [25]	To explore the quality of sleep among renal transplant patients and relevant psychosocial factors.	*n* = 438, aged > 18 years. China.	A cross-sectional study.	III	4	Patients post-renal-transplantation had emotional-psychological issues, such as anxiety, depression, and worries about rejection and graft loss and concerns about family economic burdens. Renal transplant patients’ sleep quality was lower compared to the general population, which was associated with poor well-being and QoL.
6. Trevizan et al. [28]	To discover mental disorders and symptoms experienced by heart transplant patients post-operation.	*n* = 33, aged 30–71 years. Brazil.	A cross-sectional and descriptive study.	III	3	Symptoms of depression and anxiety were discovered in most patients. The most-used treatment strategy was problem-focused coping strategies, followed by a focus on religiosity or fanciful thinking and social support. Men tended to use problem-focused strategies and women religious/fantasy practices.
7. Low et al. [19]	To explore the stressors associated with life after kidney transplantations.	*n* = 25, aged 26–72 years. Australia.	A descriptive, exploratory study.	III	N/A	Patients experienced distress caused from the transplant regimen and fear of graft rejections, infections, and the possibilities of developing cancer. Patients used coping resources, including changing their mindsets and searching for social support.
8. Grumme & Gordon [34]	To discuss the use of social media as potential supporting resources for transplant recipients.	*n* = 126 (online postings) from 58 members of an international transplant community. U.S.	A descriptive, qualitative study.	III	N/A	Two major themes were sharing overwhelming gratitude and finding sanctuary. The results suggest that social media support sites can serve as a supportive resource into the world of transplant recipients.
9. Lundmark et al. [32]	To develop Allvin et al.’s concept analysis and to identify the recovery trajectories of recovery after lung transplantation.	*n* = 15, aged 26–70 years. Sweden.	A deductive, retrospective interview study.	III	N/A	Managing symptoms; the adaptation of physical restraints, social relationships, and daily occupation; achieving psychological well-being; and emotional transition were experienced during the post-transplant recovery process. All participants endeavored to become independent during the social recovery period.
10. Schmid-Mohler et al. [21]	To discover patients’ self-management tasks in the early post-kidney transplant period.	*n* = 12, aged 42–65 years. Switzerland.	Mixed-method study using semi-structured interviews and a structured questionnaire.	III	N/A	Patients experienced difficulties in managing instability, emotions such as uncertainty and frustration, and changes in self-perception relating to relationships. Focusing on emotional management tasks and restoring stability for patients are essential for self-management.
11. White-Williams et al. [29]	To investigate the relationship between postoperative social support satisfaction, HRQoL, and survival for heart transplant recipients.	*n* = 555, mean age = 53.8 years. U.S.	A retrospective and statistical analysis.	III	4	At five and ten years post-transplantation, patients were not satisfied with their emotional support and tangible support, such as financial assistance, chores, and child and elder care. Social support satisfaction is important for the HRQoL of heart transplant recipients.
12. Burkhalter et al. [15]	To explore renal transplant recipients’ self-reported sleep disturbances.	*n* = 164, mean age = 59.1 ± 11.6 years. Switzerland.	A sequential, cross-sectional study.	III	3	Results showed a high insomnia prevalence, which had a negative impact on patients’ daytime functionality.
13. Forsberg et al. [33]	To examine the psychometric traits of organ transplant recipients and develop a measure of their symptoms and well-being.	*n* = 185, aged 19–65 years. Sweden.	A cross sectional survey.	III	2	The most disruptive problem was sleeping, followed by fatigue and muscle and joint pain, which caused distress. Patients aged < 50 years reported significant irritation and aggressive mood problems, as well as heartburn problems.
14. Urstad et al. [23]	Use an educational intervention to test the efficacy on renal recipients’ knowledge, compliance, self-efficacy, and quality of life.	*n* = 139, aged 21–76 years. Norway.	A randomized controlled study	II	6	A structured, tailor-made, postoperative educational intervention benefited patients’ compliance, self-efficacy, and mental QoL, which can increase renal recipients’ knowledge about post-transplant life.
15. Korus et al. [18]	To study the information needs of renal transplant adolescents.	*n* = 8, aged 13–17 years. Canada.	A qualitative, descriptive study.	III	N/A	Four major stressors, including body image changes, desiring to be normal, pain, and communication breakdown were identified. Coping strategies used were knowledge of the transplantation process and understanding through social support.
16. Chen et al. [16]	To quantify the amount and causes of stress experienced by kidney transplant recipients.	*n* = 153, mean age = 41.5 years. Taiwan.	A cross-sectional, descriptive design.	III	3	Patients experienced stresses, including uncertainty, limitations, complications, and difficult social interactions. Improving recipients’ self-efficacy may reduce stress. Men felt more stressed than women about interactions and uncertainty.
17. Lin et al. [5]	To investigate dark-recovery experiences, coping strategies, and the needs of adult Taiwanese heart transplant recipients.	*n* = 20, aged 32–70 years. Taiwan.	A qualitative design with retrospective data.	III	N/A	Dark recovery difficulties, including becoming a family burden, not familiar with life post-transplantation, and experiencing mental and physical discomfort and uncertainty about future were discovered. Coping strategies used were religious support, changing mindset, being positive, setting goals, and looking for a future job.
18. Myers & Pellino [35]	To determine patients’ perceptions of their own knowledge gaps and ways to enhance patients’ education.	*n* = 403, aged 19–80 years. U.S.	A descriptive, nonexperimental study.	III	3	Patients reported significantly lower scores in the QoL/psychosocial subscale. Inability to attend classes was associated with significantly lower scores in patients learning needs related to medication and follow-up care. Video tape/DVD-based teaching can be a very effective way to provide education to patients.
19. Pelgur et al. [31]	To determine the anxiety and depression levels and the training needs for liver transplant patients.	*n* = 64. Turkey.	A descriptive study.	III	2	Patients were worried about economic losses, fear or uncertainty of the future, loss of job, and changes in family relationships and role changes. Patients would like to learn issues about treatment side effects, self-care information, and infection risk information.
20. Zarifian [26]	To determine renal transplantation recipients’ symptoms and symptom distress post-operation and their relationship to QoL.	*n* = 100, aged 21–69 years. U.S.	A cross-sectional, comparative study.	III	3	Most stressful symptoms reported, including sleep problems, eating disorders, fatigue, changes in body images, and mood swings. Patients rated their quality of life as acceptable but not as good as the normal population.
21. Blanch et al. [30]	To discover psychosocial functional domains in OLT transplant recipients and the factors associated with postoperative adjustment.	*n* = 266, aged 49–62 years. Spain.	A descriptive study with structured questionnaires.	III	2	Women showed a much poorer OLT adjustment than men did. Women also showed more dysfunction in sexual and family relationships and had more psychological distress than men did.
22. Martin & Sachse [20]	To examine women’s spiritual perspectives and well-being post-kidney-transplantation.	*n* = 28, mean age = 44.36 ± 14.26 years. U.S.	A descriptive, correlational study.	III	1	Spirituality is a psychological resource that can benefit the well-being of female recipients of kidney transplants.
23. Kaba & Shanley [27]	To study the coping mechanisms used by heart transplant recipients.	*n* = 42. U.S.	A descriptive study.	III	3	Findings revealed the need for nurses to assist patients and encourage social interaction. Patients used passive appraisal coping strategies and were thus more likely to experience consequences related to stress. Additional support and encouragement should be given to patients to increase their post-transplant social contact and enhance QoL.

Note: ISM = immunosuppressive medication, QoL = Quality of Life, HRQoL = health-related quality of life, and OLT: orthotopic liver transplantation.
